# Association of intraoperative gross hematuria with acute kidney injury after cytoreductive surgery

**DOI:** 10.1515/pp-2021-0145

**Published:** 2022-02-18

**Authors:** Yumi Mitani, Yohei Arai, Tomohiro Mitani, Yoshimasa Gohda, Hideaki Yano, Isao Kondo, Emi Sakamoto, Daisuke Katagiri, Fumihiko Hinoshita

**Affiliations:** Department of Nephrology, National Center for Global Health and Medicine, Tokyo, Japan; Department of Surgery, National Center for Global Health and Medicine, Tokyo, Japan; Department of Nephrology, Graduate School of Medical and Dental Sciences, Tokyo Medical and Dental University, Tokyo, Japan; Graduate School of Medicine, University of Tokyo, Tokyo, Japan; Department of Nursing, Faculty of Health Care and Medical Sports, Teikyo Heisei University, Tokyo, Japan

**Keywords:** acute kidney injury, cytoreductive surgery combined with hyperthermic intraperitoneal chemotherapy (CRS-HIPEC), intraoperative gross hematuria, ureter injury

## Abstract

**Objectives:**

Cytoreductive surgery combined with hyperthermic intraperitoneal chemotherapy (CRS-HIPEC) has been established in the management of peritoneal carcinomatosis. Although it is still necessary to take adequate measures against major postoperative complications including acute kidney injury (AKI), consensus is lacking on how to assess and stratify risk for patients with postoperative AKI after CRS-HIPEC. The aim of this retrospective cohort study was to investigate the association of intraoperative gross hematuria as a surrogate marker of ureter injury with postoperative AKI incidence.

**Methods:**

This retrospective cohort study investigated patients without impaired preoperative kidney function who underwent CRS-HIPEC at a single referral center, and evaluated the relationship between intraoperative gross hematuria and incidence of postoperative AKI as defined by the Kidney Disease Improving Global Outcomes practice guidelines. Logistic regression analysis was performed to calculate the odds ratio of intraoperative gross hematuria for AKI, adjusting for confounding factors and other risk factors for AKI.

**Results:**

We enrolled 185 patients (males, 37%). Twenty-five patients developed intraoperative gross hematuria. Postoperative AKI occurred in 10 (40%) of 25 patients with hematuria and 28 (17.5%) of 160 patients without hematuria. The crude odds ratio for exposure to hematuria was 3.14 (95% confidence interval, 1.30–7.60; p=0.020) for postoperative AKI. Adjusted odds ratio as estimated by multivariate logistic regression was 4.57 (95% confidence interval, 1.55–13.45; p=0.006).

**Conclusions:**

Intraoperative gross hematuria is significantly associated with postoperative AKI incidence after CRS-HIPEC.

## Highlights

– Early detection and treatment of postoperative acute kidney injury is essential.
– How to prevent acute kidney injury after cytoreductive surgery is unclear.
– Intraoperative hematuria is related to postoperative acute kidney injury.
– Hematuria is easily detectable and may reflect ureter injury.
– This may improve long-term outcomes for patients undergoing cytoreductive surgery.

## Introduction

Peritoneal carcinomatosis (PC) refers to intraperitoneal dissemination of any form of cancer that originates from not only the peritoneum itself, but also metastatic carcinoma, including gastrointestinal and gynecological cancers [1]. Although PC had been considered incurable because of the difficulty of complete resection and low sensitivity to chemotherapy as recently as a few decades ago, the new treatment modality of cytoreductive surgery (CRS) combined with hyperthermic intraperitoneal chemotherapy (CRS-HIPEC) has drastically improved prognosis for patients with PC and has been accepted worldwide [2], [3], [4], [5], [6], [7], [8]. However, therapeutic methods and management of postoperative complications such as intra-abdominal abscesses, pulmonary complications, anastomotic leakage, and acute kidney injury (AKI) remain to be established [2, 9], [10], [11].

AKI is one of the most important complications, directly associated with poor prognosis [12], [13], [14]. Early detection and treatment of AKI is essential to improve patient outcome [15, 16]. Because postoperative AKI after CRS-HIPEC occurs in up to 22% of patients [17], a reliable predictor of postoperative AKI is needed. Although only a handful of studies have evaluated risk factors for postoperative AKI after CRS-HIPEC [18], those reports showed inconsistent results.

The present study focused on ureter injury as an important risk factor for postoperative AKI after CRS-HIPEC. Moreover, we supposed that intraoperative hematuria caused by ureter injury might offer a useful predictor of this AKI. In fact, ureter injury increases risk of postoperative AKI after general surgery in the fields of urology and gynecology [19, 20]. Some reports demonstrated intraoperative ureter injury in about 2–7% of patients during CRS-HIPEC [21, 22].

Whether intraoperative ureter injury is associated with postoperative AKI after CRS-HIPEC remains unknown. As ureter injury is a candidate cause of AKI that might be avoided, we decided to examine the association between ureter injury and postoperative AKI. Here, we conducted a retrospective cohort study to investigate the association between intraoperative gross hematuria as a surrogate marker of ureter injury and postoperative AKI incidence after CRS-HIPEC.

## Materials and methods

### Study design

This retrospective cohort study investigated patients who underwent CRS-HIPEC at a single referral center, the Center Hospital of the National Center for Global Health and Medicine. Ethics Board approval was attained for the study. Because of the retrospective, observational nature of this study, the committee waived the need for written informed consent. We displayed an opt-out consent document in the outpatient department of our hospital for 2 years and any patient who chose to opt out from use of their anonymized data in our study by viewing these public documents would be excluded. No patients were excluded in this manner.

Inclusion criteria were as follows: (1) patients ≥20 years old who had preoperative estimated creatinine clearance ≥50 mL/min/1.73 m^2^ without receiving renal replacement therapy; (2) patients who underwent CRS-HIPEC for primary advanced or recurrent PC from various forms of disseminated carcinomatosis between March 2010 and February 2019. However, patients who underwent CRS-HIPEC between November 2014 and February 2017 were not enrolled because they had participated in another study in advance [23]. Exclusion criteria were defined as follows: (1) patients without complete perioperative data about intraoperative gross hematuria; (2) patients receiving pre- or intra-operative prophylactic ureteric stenting or patients without perioperative data about ureteric stenting, because ureteric stenting may affect AKI development [24] as well as hematuria [25].

### Surgical technique and postoperative management

The aim of CRS surgery is to obtain complete macroscopic cytoreduction [26]. After the same preoperative management, CRS surgery was performed by the same experienced surgical team from the Department of Surgical Oncology. Peritoneal cancer index (PCI) was used to assess the extent of carcinomatosis. Surgeons routinely performed right and left parietal peritonectomies to access to the retroperitoneum and locate the ureters and major lower limb vessels. Peritoneal resection was continued by dissecting the urinary bladder peritoneum and if possible the recto-vesical peritoneum or rectal resection, if the disease had heavily infiltrated into the rectum and/or sigmoid. In females, the uterus and both ovaries, if not previously resected, were generally removed by total abdominal hysterectomy and bilateral salpingo-oophorectomy. The greater and lesser omentum and falciform ligament were resected. The appendix was removed by appendectomy, cecectomy, or ileocecal resection, as required. Surgeons mobilized the liver to facilitate right diaphragmatic peritonectomy and stripped the hepato-duodenal ligament of the peritoneum. If necessary, the gallbladder, left diaphragmatic peritoneum, and left and right liver capsules were resected. Further visceral resection was a combination of splenectomy, left diaphragmatic peritoneal resection and distal or total gastrectomy, depending on the extent of the disease.

Following CRS, HIPEC was carried out using an open coliseum technique for 60 min at a temperature of 42–43 °C. Chemotherapy regimens were determined according to the primary disease: mitomycin-C for pseudomyxoma and endometrial cancer-derived peritoneal metastases; cisplatin and doxorubicin for malignant peritoneal mesothelioma; and oxaliplatin for colorectal peritoneal carcinomatosis. Other drugs were administered according to the choice of the surgeon, taking into account patient allergies to standard drugs, recurrence, and financial constraints. Three patients did not undergo HIPEC due to financial constraints, as HIPEC is not covered by insurance in Japan.

After CRS-HIPEC, most patients were slated for early postoperative intraperitoneal chemotherapy (EPIC) over a period of four days, from day 1 to day 4 after surgery. However, 48 patients did not undergo EPIC because of the postoperative course, such as unstable hemodynamics, bleeding, and abnormal results of blood tests. Postoperative management such as infusion and blood transfusion was also changed depending on the patient’s condition.

### Exposure and outcome

We defined intraoperative gross hematuria as exposure and postoperative AKI as primary outcome. Intraoperative gross hematuria was diagnosed by nurses and was reported in surgical nursing records.

AKI diagnosis and severity stage were based on serum creatinine, in accordance with the Kidney Disease Improving Global Outcomes (KDIGO) practice guidelines [27]. AKI was defined as an increase to at least 1.5 times baseline or an increase of ≥0.3 mg/dL in serum creatinine from baseline within 7 days after surgery. The staging system for evaluating severity of AKI was also in accordance with KDIGO practice guidelines.

### Variables for adjustment

In this analysis, we selected three confounding factors (sum of sub scores for abdominoperitoneal regions 4–8 (4, left flank; 5, left lower; 6, pelvis; 7, right lower; 8, right flank) for PCI score, body mass index (BMI), and estimated blood loss) and four risk factors for postoperative AKI (age, preoperative eGFR, platinum-based infusion (cisplatin) and PCI score). Reasons for the selection of these variables are described in the Discussion.

### Data collection

We extracted the following preoperative variables from electronic medical records: demographics (age, sex, BMI), type of cancer, American Society of Anesthesiologists (ASA) physical status, PCI score, preoperative laboratory data (eGFR, creatinine, urea, hemoglobin, platelet, and albumin), chronic comorbidities such as hypertension and diabetes mellitus, and perioperative medications (angiotensin-converting enzyme inhibitors, angiotensin II receptor blockers, diuretics, nonsteroidal anti-inflammatory drugs, and pregabalin).

We extracted the following operative variables from electronic medical records: operative methods (operation time and anesthesia time), type of drugs for HIPEC and EPIC, and operative findings (gross hematuria, estimated blood loss, urine output, and transfusion volume). Preoperative serum creatinine and postoperative serum creatinine at several times within seven days after surgery were collected. The frequency of blood tests was determined by surgeons according to the clinical courses of patients.

### Statistical methods

Crude odds ratio and 95% confidence interval (CI) were calculated and tested with the χ^2^ test with Yates correction. To adjust the confounding and random bias of risk factors, we estimated the adjusted odds ratio of hematuria for AKI by multivariate logistic regression with linear terms of confounding factors and risk factors without any interaction terms. Our missing data analysis procedures used missing at random (MAR) assumptions. We used the “mice” package in R to make 20 copies of data and to impute missing values by a fully conditional specification (FCS) algorithm. We independently analyzed the 20 copies of data in the multivariate logistic regression analysis. Estimates of the odds ratio were averaged to yield a single mean estimate and 95% CI according to Rubin’s rules [28].

Values of p<0.05 were considered statistically significant. All statistical procedures were performed on computing environment R version 3.5.3.

## Results

**Figure 1:**
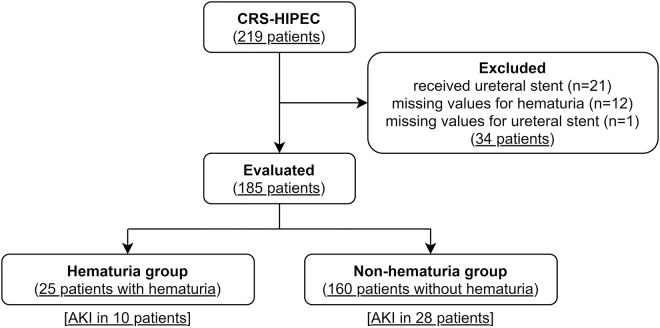
Flow diagram of the study population. CRS-HIPEC, cytoreductive surgery plus hyperthermic intraperitoneal chemotherapy; AKI, acute kidney injury.

The study selection process is displayed in (Figure 1). A total of 219 patients who underwent CRS-HIPEC were identified in the electronic database. Among these patients, data on prophylactic ureteric stenting were missing for 1 patient, data on intraoperative gross hematuria were missing for 12 patients and 21 patients underwent pre- or intraoperative prophylactic ureteric stenting. As a result, 185 patients were included in this retrospective study.

The demographic parameters are detailed in (Table 1). The ratio of males was higher in the non-hematuria group than in the hematuria group. Age and BMI were similar between groups. Preoperative laboratory data including eGFR were also similar between groups.

**Table 1:** Baseline characteristics of participants.

	Overall	Hematuria group	Non-hematuria group
	(n=185)	(n=25)	(n=160)
Demographics			
Age, years	57 (47–66)	54 (48–63)	57 (47–66)
Male sex	68 (37)	4 (16)	64 (40)
BMI, kg/m^2^	22.4 ± 3.57	20.0 ± 3.6	22.7 ± 3.4
Obesity (BMI>30 kg/m^2^)	4 (2.1)	0	4 (2.5)
Primary tumor site			
PMP	136 (73.5)	21 (84)	115 (72)
Malignant mesothelioma	7 (3.7)	0 (0)	7 (4.3)
Colorectal/appendiceal cancer	41 (22.2)	4 (16)	37 (23.1)
Endometrial cancer	1 (0.5)	0 (0)	1 (0.6)
Others			
PCI score	20.44 ± 10.29	18 ± 10.63	20.82 ± 10.19
PCI score 4–8	9.87 ± 4.88	9.31 ± 5.52	9.95 ± 4.77
Preoperative complication status			
ASA score			
≤2	173 (93.5)	24 (96)	149 (93.1)
3	12 (6.5)	1 (4)	11 (6.9)
Preoperative laboratory data			
eGFR (mL/min/1.73 m^2^)	82.44 ± 19.81	78.33 ± 17.50	83.08 ± 20.08
Serum creatinine, mg/dL	0.68 ± 0.17	0.67 ± 0.16	0.68 ± 0.17
BUN, mg/dL	13.80 ± 4.31	14.36 ± 5.33	13.70 ± 4.13
Hemoglobin, g/dL	12.42 ± 1.59	11.96 ± 1.63	12.50 ± 1.57
Platelets, ×10^4^/μL	26.95 ± 9.00	29.10 ± 9.11	26.62 ± 9.11
Albumin, g/dL	3.86 ± 0.54	3.72 ± 0.52	3.88 ± 0.54
Comorbidities			
Diabetes	13 (7)	0 (0)	13 (8.1)
Hypertension	34 (18.4)	3 (12)	31 (19.3)
Medications			
ACEIs/ARBs	19 (10.2)	1 (4)	18 (11.2)
Diuretics	5 (2.7)	3 (12)	2(1.2)
NSAIDs	10 (5.4)	4 (16)	6 (3.8)
Pregabalin	0	0	0

Characteristics of the 185 eligible patients. Continuous variables are presented as mean ± standard deviation or median (interquartile range). Categorical variables are presented as numbers (percentages). Conversion factors for units: Cr in mg/dL to mmol/L, ×88.4; eGFR in mL/min/1.73 m^2^ to mL/s/1.73 m^2^, ×0.01667. BMI, body mass index (calculated as weight in kilograms divided by height in meters squared); PMP, pseudomyxoma peritonei; PCI, peritoneal carcinomatosis index; PCI score 4–8: sum of sub scores for abdominoperitoneal regions 4–8 (4, left flank; 5, left lower; 6, pelvis; 7, right lower; 8, right flank) for PCI; ASA, American Society of Anesthesiologists; eGFR, estimated glomerular filtration rate; BUN, blood urea nitrogen; ACEI, angiotensin-converting enzyme inhibitor; ARB, angiotensin II receptor blocker; NSAIDs, nonsteroidal anti-inflammatory drugs.

Operative data are detailed in (Table 2). Patients in the hematuria group had greater estimated blood loss and transfusion volumes than those in the non-hematuria group. The other variables were considered similar between the non-hematuria and hematuria groups. PCI scores were missing in 66 patients (33%); 52 in hematuria group and 14 in non-hematuria group. Otherwise, only 1 albumin value was missing.

**Table 2:** Operation data.

	Overall	Hematuria group	Non-hematuria group
	(n=185)	(n=25)	(n=160)
Operative methods			
Operation time, h	10.91 ± 2.45	11.69 ± 3.00	10.79 ± 2.39
Anesthesia time, h	13.00 ± 2.54	13.66 ± 3.00	12.89 ± 2.44
Intraoperative chemotherapy			
HIPEC	182 (98.3)	25 (100)	157 (98)
Mitomycin	144 (77.8)	23 (92)	121 (75.6)
Adriamycin + cisplatin	8 (4.3)	0	8 (5)
Oxaliplatin	27 (14.6)	2 (8)	25 (15.6)
EPIC	137 (74)	21 (84)	116 (72.5)
5FU	130 (70.2)	21 (84)	109 (68.1)
Adriamycin + cisplatin	6 (3.2)	0	6 (3.8)
Operative finding			
Estimated blood loss, mL	546 (290–1,357)	724 (332–1724)	516 (290–1,349)
Urine output, mL/kg/h	1.64 ± 1.25	1.97 ± 1.25	1.59 ± 1.25
Transfusion volume, mL	2,760 (1720–3,760)	3,230 (1910–4,760)	2,740 (1,698–3,720)
Red cell concentrate (units)	6 (4–10)	8 (4–14)	6 (4–10)
Plasma, units	16 (12–20)	16 (12–26)	16 (10–20)
Platelets, units	2.88 ± 8.83	2.8 ± 6.65	2.89 ± 9.12
Infusion volume, mL	8,450 (6,650–11,950)	8,450 (6,300–13,640)	8,475 (6,650–11,875)

Continuous variables are presented as mean ± standard deviation or median (interquartile range). Categorical variables are presented as number (percentage). HIPEC, hyperthermic intra-peritoneal chemotherapy; EPIC, early postoperative intra-peritoneal chemotherapy; 5FU, fluorouracil.

**Figure 2:**
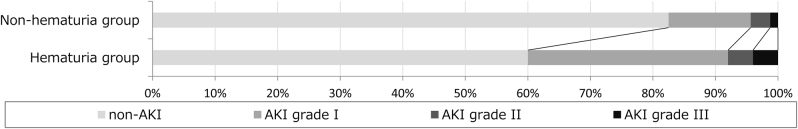
Incidence of AKI among patients with and without intraoperative gross hematuria. AKI, acute kidney injury.

Incidence rates of AKI are detailed in (Figure 2). Of the 185 patients in our study, postoperative AKI occurred in 38 (20.5%) and temporary hemodialysis was performed for two patients (2 of 185 patients, 1.0%). AKI developed in 40% of patients in the hematuria group (10 of 25 patients), and in 17.5% of patients in the non-hematuria group (28 of 160 patients). In the hematuria group, eight patients (32%) developed AKI grade 1, 1 patient (4%) developed AKI grade 2, and 1 patient (4%) developed AKI grade 3. In the non-hematuria group, 21 patients (13%) developed AKI grade 1, 5 patients (3%) developed AKI grade 2, and 2 patients (1%) developed AKI grade 3 and required temporary hemodialysis. Of the two patients who required hemodialysis, one was diagnosed to be due to non-hemorrhagic hypovolemia and platinum-based infusion (cisplatin), and the other to be due to hemorrhage. In both cases, clear evidence of ureteral injury was not detected. In the entire study, there were no case of ureteral injury which required additional treatment such as ureteric stenting and ureteric reconstruction. The crude odds ratio for exposure to hematuria was 3.14 (95% CI, 1.30–7.60; p=0.020) for postoperative AKI. Adjusted odds ratio estimated by multivariate logistic regression was 4.57 (95% CI, 1.55–13.45; p=0.006). The adjusted covariates were sum of sub scores for abdominoperitoneal regions for PCI score, BMI, estimated blood loss, age, preoperative eGFR, platinum-based infusion (cisplatin) and PCI score. Those results were statistically significant.

## Discussion

This is the first study to investigate the association between exposure to intraoperative gross hematuria and postoperative AKI incidence after CRS-HIPEC. Multivariate logistic regression analysis showed that the odds ratio for intraoperative gross hematuria was independently associated with postoperative AKI incidence after CRS-HIPEC.

Only a few studies have evaluated the actual incidence and risk factors of postoperative AKI after CRS-HIPEC [17]. Previous reports have shown inconsistent results and integration of these results was difficult due to small and non-homogeneous patient populations, different cancer staging criteria, different scopes of CRS and different therapeutic approaches. In the largest study (n=475), Cata et al. [17] reported age, obesity (BMI >30 kg/m^2^), preoperative pregabalin, cisplatin, estimated blood loss, and splenectomy as risk factors for postoperative AKI after CRS-HIPEC. Regardless of CRS-HIPEC, many studies about general surgery have reported lower eGFR itself as a risk factor for AKI [29]. A previous study about CRS-HIPEC also reported eGFR as a risk factor for postoperative AKI [30].

As ureteral injury is known to be a factor causing postoperative AKI in abdominal surgery [19], we decided to investigate whether ureteral injury is also a risk factor for postoperative AKI in CRS-HIPEC. We hypothesized that intraoperative gross hematuria was mainly caused by some kind of ureter injury during CRS-HIPEC and regarded intraoperative gross hematuria as a surrogate marker for intraoperative ureter injury. Although the exact pathophysiology of the association between ureter injury and AKI was unclear, Siddighi et al. [19] hypothesized that the increase in serum creatinine was caused by varying degrees of ureteral obstruction resulting in increased ureteral pressure proximal to the point of obstruction, leading to elevated intrarenal pressures and transient dysfunction. Presently, no gold standard for diagnosing ureter injury is available. Although intraoperative cystoscopy [35] and postoperative CT [31] reportedly allow precise diagnosis of unsuspected ureteral injuries, it remains unclear whether these tests are able to detect varying degrees of ureteral obstruction as suggested by Siddighi [19]. In our hospital, intraoperative cystoscopy, postoperative CT and ultrasonography were not routinely performed in CRS-HIPEC, and this might also be the case in other hospitals. The clinical data on ureteral obstruction or stenosis were also not available in our study. Hematuria, fever, flank or groin pain, or vaginal urinary leakage have been reported as signs and symptoms of ureter injury [31]. However, we think these symptoms are not direct evidence of ureter injury, because they may be influenced by intraoperative drugs and inflammation from invasive procedures. On the other hand, intraoperative gross hematuria is supposed to directly reflect the effects of ureter injury. The incidence ratio of gross hematuria was much higher than the incidence ratio of ureter injury reported in previous studies, at 2–7% [21, 22]. We think that observation of intraoperative gross hematuria is more suitable for detecting minor ureter injury without urinary stenosis or obstruction compared to the imaging studies used to diagnose ureter injury in previous studies, and thus may be more versatile in clinical practice.

The reasons for selecting the confounders and significant risk factors to be adjusted are as follows. As risk factors of intraoperative ureteric injury, Brandes et al. [31] reported that intraoperative hemorrhage, such as sudden hemorrhage, as well as greater blood loss, long operative time, large retroperitoneal mass and more transfusions were associated with ureter injury. Abu-Zaid et al. [32] reported that patient body habitus, clinical diagnosis, size and extent of the mass, skill and experience of the surgeon, and the past surgical history of the patient influence the risk of operative ureteric injury . For large retroperitoneal masses and extension of the mass, we substituted these for the total of sub scores for abdominoperitoneal regions 4–8 (4, left flank; 5, left lower; 6, pelvis; 7, right lower; 8, right flank) for PCI score. PCI score was also reported as a risk factor for postoperative AKI after CRS-HIPEC [33, 34].

Some known risk factors of postoperative AKI could not be adjusted in our study. Few obese patients were included in the present study and no participants received pregabalin. Further, data were lacking on history of procedures such as splenectomy and past surgical history. According to such baseline characteristics and aforementioned research, we selected three confounding factors (sum of sub scores for abdominoperitoneal regions 4–8 (4, left flank; 5, left lower; 6, pelvis; 7, right lower; 8, right flank) for PCI, BMI, and estimated blood loss) and four risk factors for postoperative AKI (age, preoperative eGFR, platinum-based infusion (cisplatin) and PCI score).

Although there were no obvious differences in most parameters between the hematuria and non-hematuria groups, patients in the hematuria group had greater estimated blood loss and transfusion volumes than those in the non-hematuria group (Tables 1 and 2). This may suggest that the hematuria group had more invasive surgery than the non-hematuria group.

The result of multivariate logistic regression analysis showed that intraoperative gross hematuria was independently associated with the incidence of postoperative AKI after CRS-HIPEC, suggesting that minor ureteral injury can cause postoperative AKI. Therefore, when intraoperative gross hematuria is observed, it may be possible to take measures to prevent AKI, which may improve the prognosis. In addition, this study may lead to the clarification of the mechanism of intraoperative gross hematuria and may clarify the optimal intraoperative and postoperative management.

Adequate fluid intake and urethral catheter are recommended to prevent urinary retention and obstructive uropathy due to clot formation [36] when the possibility of ureter injury is detected by observation of intraoperative gross hematuria. Moreover, adequate blood transfusion to correct coagulopathies, tranexamic acid, and alkalinizing urine therapy could be used to prevent urinary obstruction, because these treatments are generally applied to gross hematuria in patients with autosomal dominant polycystic kidney disease [37]. In CRS-HIPEC, the role of these treatments is a topic for future study. On the other hand, the use of prophylactic ureteral stenting has been advocated to facilitate localization of ureters and immediately recognize any ureter injury. However, Taryn et al. identified ureteral stenting as a risk factor for postoperative AKI following colon or rectal resection [24]. Establishment of an accurate selection of patients who could benefit from pre- or intraoperative prophylactic ureteric stenting before CRS HIPEC is thus necessary [38].

We recommend that surgeons increase the frequency of blood testing and avoid exposure to nephrotoxic drugs and agents, in expectation of the appearance of postoperative AKI, if intraoperative gross hematuria is observed. Future investigations should determine what kinds of surgical techniques are needed to prevent ureter injury leading to AKI without affecting surgical removal of the tumor, and what measures offer the best outcomes after hematuria occurs. We also think that aggressive CRS-HIPEC should not be avoided, to improve the prognosis of peritoneal carcinomatosis. However, postoperative AKI might unfavorably influence the prognosis and surgical outcomes of CRS in a proportion of patients with peritoneal carcinomatosis. We therefore suggest that surgeons pay attention to ureter injury and intraoperative gross hematuria causing postoperative AKI in this aggressive surgery.

Various limitations to the present study need to be considered when interpreting the results. First, multivariate logistic regression can only account for measured or known confounders. Second, this was a single-center study and the results thus need to be validated in multicenter trials. Third, this study included some measurement errors for hematuria and AKI. However, no differences in detection rates of AKI were evident between intraoperative gross hematuria and non-hematuria groups since there were no differences in the surgical methods or frequency of bloods tests, even if intraoperative gross hematuria was present. Fourth, the rate of incomplete data for PCI score was relatively high (n=64; 34.6%), which was the important factor.

## Conclusions

The incidence of intraoperative gross hematuria was found to be significantly associated with postoperative AKI, implying that ureter injury causes postoperative AKI. Complete examination of the mechanisms causing intraoperative gross hematuria and clarification of optimal postoperative management may improve long-term outcomes for patients undergoing CRS-HIPEC.
